# Megakaryocytic Expansion in Gilteritinib-Treated Acute Myeloid Leukemia Patients Is Associated With AXL Inhibition

**DOI:** 10.3389/fonc.2020.585151

**Published:** 2020-12-09

**Authors:** Kran Suknuntha, Yoon Jung Choi, Ho Sun Jung, Aditi Majumder, Sujal Shah, Igor Slukvin, Erik A. Ranheim

**Affiliations:** ^1^ Department of Pathology and Laboratory Medicine, University of Wisconsin, Madison, WI, United States; ^2^ Wisconsin National Primate Research Center, University of Wisconsin, Madison, WI, United States; ^3^ Chakri Naruebodindra Medical Institute, Faculty of Medicine, Ramathibodi Hospital, Mahidol University, Samut Prakan, Thailand

**Keywords:** gilteritinib (Xospata), Flt3, internal tandem duplication (ITD), AXL, megakaryocyte, AML—acute myeloid leukemia, midostaurin

## Abstract

Numerous recurrent genetic mutations are known to occur in acute myeloid leukemia (AML). Among these common mutations, Fms-like tyrosine kinase 3 remains as one of the most frequently mutated genes in AML. We observed apparent marrow expansion of megakaryocytes in three out of six patients with Flt3-mutated AML following treatment with a recently FDA-approved Flt3 inhibitor, gilteritinib which possesses activity against internal tandem duplication and tyrosine kinase domain Flt3 mutations and also inhibits tyrosine kinase AXL. To assess whether biopsy findings can be attributed to promotion of megakaryocytic (Mk) differentiation with gilteritinib, we devised a cellular assay by overexpressing double mutated Flt3-ITD^Y591F/Y919F^ in chronic myeloid leukemia cell line K562 to study Mk differentiation in the presence of Flt3 and AXL inhibitors with non-mutually exclusive mechanisms. These experiments demonstrated the lack of direct effect Flt3 inhibitors gilteritinib and quizartinib on megakaryocytic differentiation at either transcriptional or phenotypic levels, and highlighted antileukemic effects of AXL receptor tyrosine kinase inhibitor and its potential role in megakaryocytic development.

## Introduction

Fms like tyrosine kinase 3 (Flt-3), also known as fetal liver kinase-2 (Flk2), is a receptor type tyrosine kinase encoded by the *Flt3* gene. Flt3/Flk-2 ligand is a hematopoietic cytokine that plays an important role as a co-stimulatory factor in the proliferation, differentiation, and survival of hematopoietic stem and progenitor cells, including megakaryocyte/erythroid cells (1), and in the development of the immune system (2). During hematopoiesis, megakaryocytes (Mk) develop from hematopoietic stem cells (HSCs) under tight control of Wnt/β-catenin signaling (3, 4). Seo and colleagues demonstrated that Wnt signaling inhibitor, Wnt-C59, and Flt3 inhibitor, TCS 359, significantly increased platelet-like particle production from Mk supporting the crucial role of Wnt signaling pathway and Flt3 in Mk development and maturation (5).

A Flt3 internal tandem duplication (ITD) mutation disrupts the autoinhibitory function of the receptor kinase domain, resulting in constitutive autophosphorylation of Flt3. Flt3-ITD is a common driver mutation present in approximately a quarter of adult AML cases (6) and confers significant negative prognostic impact on patients with AML (7, 8). The sizes of ITD vary dramatically from case to case (9). The ITD consists of in-frame insertions of duplicated sequences in a triplet. The duplicated sequences are mostly located in the juxtamembrane region, while the remaining are located in tyrosine kinase domain (TKD) (10, 11).

In 2018, FDA approved gilteritinib for the treatment of relapsed or refractory Flt3-mutated AML. Gilteritinib is a potent and highly selective Flt3 inhibitor with activity against both Flt3-ITD and TKD. It also inhibits the tyrosine kinase AXL, which is implicated in Flt3 inhibitor resistance (12). In a phase 3 randomized control trial, gilteritinib was superior to chemotherapy in achieving complete remission with full or partial hematologic recovery (13). In a retrospective analysis (14), marked myeloid differentiation with granulocytic hyperplasia occurred in 47.6% of patients (10 out of 21 patients) treated with gilteritinib consistent with gilteritinib-induced differentiation of leukemic blasts. In the present study, we describe bone marrow biopsies from six AML patients with Flt3 mutation refractory to standard regimens and receiving gilteritinib. Three patients demonstrated increased megakaryocytes while the others remained unchanged. We devised a cellular assay to address this clinical finding by overexpressing double mutated Flt3-ITD^Y591F/Y919F^ in chronic myeloid leukemia cell line K562 to study Mk differentiation in the presence of small molecule inhibitors.

## Materials and Methods

### Participants and Quantification of Megakaryocytes in the Bone Marrow Biopsy

All patients ≥18 years old who had a diagnosis of acute myeloid leukemia with confirmed genetic status of Flt3 i.e. wild-type, Flt3-internal tandem duplication (ITD), or tyrosine kinase domain mutation (TKD) during 2019–2020 at the University of Wisconsin Hospitals and Clinics were included in the study. Gilteritinib-treated patients must have at least one bone marrow biopsy before and after treatment. The study protocol was approved by institutional review board of the University of Wisconsin, Madison. The quantification of megakaryocyte was evaluated on the biopsy performing after intensive course of chemotherapy in all patients. The number of Mk were counted on the Hematoxylin & Eosin (H&E) stained biopsy slides and reported as number per low-power field (100× magnification) by blinded scientist/pathologist. All Mk were counted and divided by the total number of low-power fields assessable on the biopsies. Representative CD61 immunohistochemistry was performed on the selected cases to highlight Mk.

### Cell Culture, Small Molecules, and Megakaryocytic Differentiation

Human chronic myeloid leukemia (CML) in blast crisis cell line (K562) was procured from the American Type Culture Collection (ATCC, Manassas, VA, USA) and maintained at 37°C, 5% CO2 in complete medium, containing Iscove’s Modified Dulbecco’s Medium (IMDM), 1X penicillin/streptomycin (Gibco), and supplemented with 10% (v/v) fetal bovine serum (FBS).

To differentiate K562 into Mk, cells were treated with Phorbol 12-myristate 13-acetate (PMA, Sigma-Aldrich, St. Louis, MO, USA), gilteritinib, quizartinib, dubermatinib (TP-0903), and midostaurin for 48–72 h as previously described (15). Where indicated, low-serum medium supplemented with 1% FBS and 1 μM imatinib were used.

Gilteritinib, quizartinib, dubermatinib (TP-0903), and midostaurin were from Cayman chemical (Ann Arbor, MI, USA). All small molecules were dissolved in DMSO and store in −20°C. pMSCV-Flt3-ITD-Y591F/Y919F was procured from Addgene (Addgene.org). Flt3-ITD-Y591F/Y919F cassette was subcloned into lentiviral vector to produce viral particles using 293T cell.

### Gene Expression Analysis by Quantitative Real-Time PCR

A total of 5 × 10^5^ indicated K562 cells/well were plated in 12-well plates in a low-serum medium with 1 μM imatinib and 5 nM PMA. Cells were treated with 100 nM gilteritinib for 24 h before harvesting. Cells from three independent experiments were pooled and subsequently RNA extracted using PureLink RNA micro kit (Life Technologies) with on-column DNase treatment. cDNA was synthesized from 0.5 μg of RNA using QuantiTect Reverse Transcription kit (Qiagen) and self-designed specific primers column (Life Technologies). qPCR was performed using SensiFAST SYBR (Bioline) and self-designed specific primers specific for Mk lineage including *SCL*, *FLI1*, *GATA1*, and glyceraldehyde 3-phosphate dehydrogenase *(GAPDH)* genes (**Supplement Table 1** **)**. Reactions were carried out in duplicates on Mastercycler realplex thermal cycler (Eppendorf) and relative fold change in the target gene expression levels were calculated by minimal cycle threshold values (Ct) normalized to the reference expression of *GAPDH* in each sample.

### Cell Proliferation Assays

K562 was cultured in 24-well plates at 2 × 10^4^ cells/well in the presence of gilteritinib, quizartinib, and dubermatinib (TP-0903) for 72 h. Viable cell count was performed using propidium iodide method on Cellometer Auto2000 (Nexcelom Bioscience).

### Western Blot Assay

Cell were suspended in whole cell lysis buffer (1% SDS and 60 mM Tris-Cl, pH 6.8) with phosphatase inhibitor cocktails and protease inhibitor cocktail tablet (Sigma). Cell lysates (20 µg) were separated by Mini-protean TGX gels (Bio-Rad). The separated proteins were transferred to a PVDF membrane and were stained with antibodies for p-Flt3 (Cell Signaling), Flt3 (Cell Signaling), and GAPDH (SantaCruz). Immunoblots were visualized using the ECL PLUS detection kit (Amersham Pharmacia) and analyzed using ChemiDox XRS+ Image Lab Software Version 5.2.1 (Bio-Rad).

### The Concentration-Response Assay

K562 was cultured in 96-well plates in duplicates containing various concentrations of gilteritinib ranging from 4 nM to 4 μM. After 24 h of culture, viable cells were measured by Cell Titer Blue (Promega) using Spectramax i3x (Molecular Device) on fluorescent mode. A sigmoidal dose-response curve was fitted to the cell proliferation data from three independent experiments using GraphPad software (GraphPad, San Diego, CA, USA).

### Flow Cytometry

Cells were harvested and stained with Ghost Dye™ (Tonbo Biosciences, Madison, WI, USA), CD41a, and CD61 (BD Bioscience) for 30 min at 4°C. Cells were washed before analyses. Untreated cells were included to establish a threshold for positive gating. For apoptosis assay, externalization of phosphatidyl serine, the characteristic feature of apoptotic cells was determined by Annexin V staining. Following treatment with inhibitors for 48–72 h, cells were washed twice with annexin V binding buffer. Cells were then stained with annexin-V–PE and 7-aminoactinomycin D (7-AAD) using the annexin V-PE Apoptosis Detection Kit (BD Bioscience) according to the manufacturer’s protocol. Isotype-matched controls were used to set the threshold for non-specific background staining. All flow cytometric analysis was performed on MACSQuant Analyzer 10 (Miltenyi Biotec Inc., San Diego, CA, USA). The data was analyzed using FlowJo software (Tree Star, Inc., Ashland, OR, USA).

### Statistical Analysis

Data obtained from multiple experiments were reported as mean ± SD. Significant levels were determined by two-way ANOVA followed by Dunnett’s multiple comparison or controlling the false discovery rate, Fisher’s exact test, or *t-*test, two-tail as appropriate. *p* < 0.05 was considered significant. All the graphs and statistics were performed using GraphPad Prism software.

## Results

### Expansion of Bone Marrow Megakaryocytes in Flt3-Mutated Acute Myeloid Leukemia

**Figure 1:**
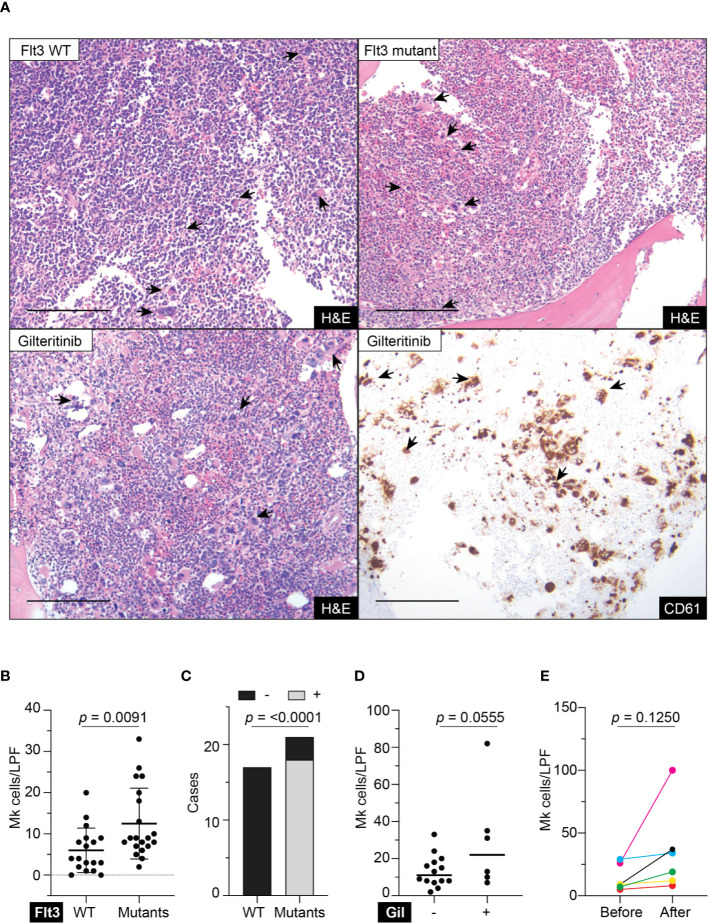
Megakaryocytic profile in acute myeloid leukemia patients. **(A)** Top panel showing H&E stains of representative trephine bone marrow biopsies from Flt3 wild type and mutant acute myeloid leukemia patients. Lower panel showing sections from the same Flt3 mutant patient showing in the upper right at 1 month following gilteritinib treatment. CD61 immunohistochemistry highlight megakaryocytes scattering in the marrow. H&E, hematoxylin & eosin; scale bar = 100 μm; arrows indicate representative megakaryocytes. **(B)** Quantification of megakaryocytes in the marrow of acute myeloid leukemia patients. Biopsies prior to gilteritinib treatment were evaluated in the gilteritinib-treated patients. (N = 38; Mk, megakaryocytes; LPF, low power field at 100× magnification). **(C)** Bar graph showing distribution of midostaurin treatment between Flt3 wild type and mutant group. – indicates without midostaurin, + indicates receiving midostaurin, WT, wild type. **(D)** Scattered plot representing megakaryocytes in the marrow of Flt3-mutated acute myeloid leukemia patients treated with or without gilteritinib. Each dot representing an individual patient. (Number of Mk was averaged from all available biopsies including pre- and post-gilteritinib treatment, N = 20; Gil, gilteritinib.) **(E)** Quantification of megakaryocytes in the marrow of Flt3-mutated acute myeloid leukemia patients before and after receiving gilteritinib.

Retrospective review of the trephine bone marrow biopsies demonstrated that Flt3-mutated AML patients have significantly increased megakaryocytes (**Figures 1A, B**). To exclude confounding bias, demographic data was analyzed (**Table 1**) and identified midostaurin therapy as a potential confounder, which exclusively distributed to the Flt3-mutated group (**Figure 1C**). Midostaurin can increases megakaryocytic differentiation in human leukemia lines (16); however, a clinical correlation has not yet been reported. Among Flt3-mutated AML patients, six patients who failed to achieve remission with standard chemotherapy including cytarabine and anthracycline agents, were given gilteritinib. Within the Flt3 mutant group, gilteritinib-treated patients tend to have increased Mk although it did not achieve statistical significance (**Figure 1D**). We further evaluated individual gilteritinib-treated patients and compared numbers of Mk in the biopsy before and after treatment. We found three out of six patients (50%) showed increased in Mk following treatment (**Figure 1E**, **Supplemental Figure 1**, and **Table 2**). Of note that two patients who had drastically increased in Mk following gilteritinib, one has mutation in the kinase domain (Asp835Tyr) and the other has Flt-ITD. Both of them have additional NPM1 and DNMT3A mutations. Given that in certain cases, marked expansion of Mk, which was not present prior to treatment, was observed (**Figure 1A**) and the limited number of cases, we devised a cellular assay to explore the potential effects of gilteritinib on Mk differentiation.

**Table 1:** Demographic information.

	FLT3 WILD TYPE(N = 18)	FLT3 MUTANT(N = 20)	P
**AGE, years**	66 ± 18	59 ± 17	*0.1075*
**GENDER, M/F**	11/7	7/13	*0.1341*
**CBC**			
** White blood cell, ×10^6^** **cells/m l**	2.0 ± 11	4.6 ± 45	*0.2091*
** Hemoglobin, g/d l**	10.7 ± 2	9.9 ± 2.6	*0.8751*
** Hematocrit, %**	30.5 ± 9	28.1 ± 10.0	*0.8591*
** Platelet, ×10^6^ cells/ml**	51.0 ± 72	47.5 ± 75	*0.9860*
**MIDOSTAURIN**	0	17	*<0.0001*
**BONE MARROW TRANSPLANT**	5	7	*0.6325*

**Table 2:** Clinical information of gilteritinib-treated Flt3-mutated leukemic patients.

AGE	GENDER	FLT3 STATUS	MUTATION	SCT	Marrow status	Prior treatment
**64**	F	TKD (D835Y)	NPM1, DNMT3A	No	**Baseline**: ~65% blasts **Best response**: hypercellular marrow with increased Mk and 27% blasts	Cytarabine+midostaurin
**45**	F	ITD	NPM1, DNMT3A	Yes	**Baseline**: 59% blasts **Best response**: hypercellular marrow with increased Mk and 17% blasts	Cytarabine+midostaurin
**60**	F	ITD	NPM1, DNMT3A	No	**Baseline**: 85% blasts **Best response**: 50–70% cellularity with increased Mk and 2% blasts, MRD positive	Daunorubicin+midostaurin
**47**	F	TKD (D835E)		Yes	**Baseline**: 2% blasts with cytogenetic evidence of residual disease **Best response**: normocellular marrow with left shift granulopoiesis, no detectable Flt3 mutants and MRD negative	Cytarabine+daunorubicin
**51**	F	ITD	NPM1	Yes	**Baseline**: 20% blasts **Best response**: hypocellular marrow and <1% blasts, MRD negative	Idarubicin+midostaurin
**65**	F	ITD		No	**Baseline**: ~95% blasts **Best response**: hypercellular marrow and 21% blasts	Cytarabine+idarubicin+ midostaurin

TKD, tyrosine kinase domain; ITD, internal tandem duplication; SCT, stem cell transplantation; MRD, minimal residual disease; Mk, megakaryocyte.

### Biological Activities of Flt3 Inhibitors on Cell Proliferation, Megakaryocytic Differentiation, and Apoptosis

**Figure 2:**
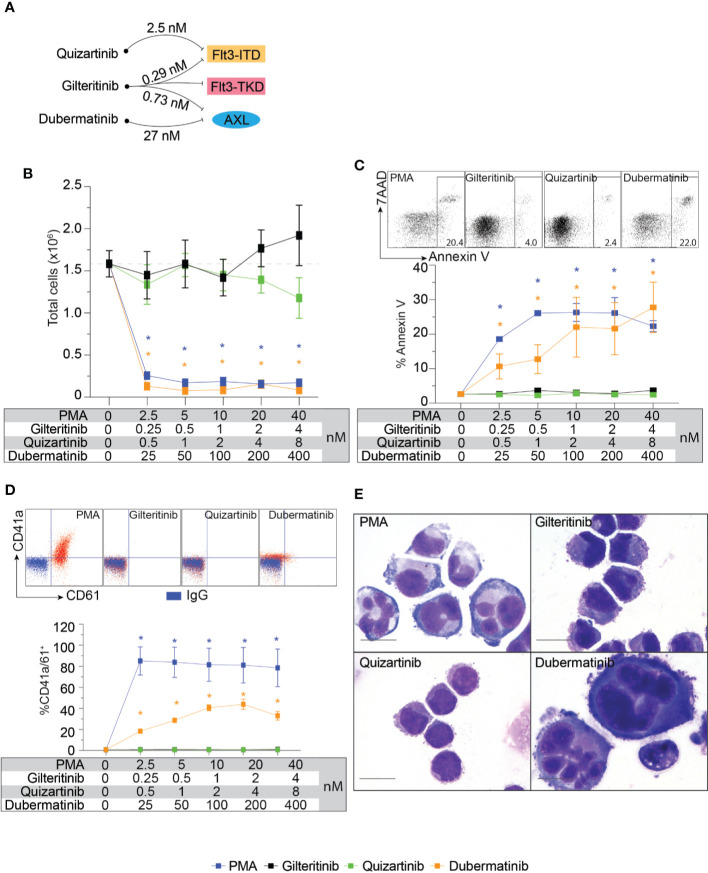
Biological effects of Flt3 and AXL inhibitors on cell proliferation, megakaryocytic differentiation, and apoptosis in K562. **(A)** Schematic diagram showing targets of drug action and approximate 50% inhibitory concentration for each target. **(B)** Cell proliferation assay showing total live cells following 72-h treatment with indicating condition in the table. Results from three independent experiments are shown as mean ± SD. * indicates significance compared with untreated (dash line). **(C)** Flow cytometric analysis of Annexin V and 7AAD following 48-h treatment with indicating condition in the table. Representative dot plots and a gating threshold are shown. Results from three independent experiments are shown as mean ± SD. * indicates significance compared with untreated. **(D)** Flow cytometric analysis of CD41a and CD61 following 72-h treatment with indicating condition in the table. Representative dot plots are shown; isotype controls are shown in blue and the treated cells are shown in red. Results from three independent experiments are shown as mean ± SD. * indicates significance compared with untreated. **(E)** Wright-Giemsa stain of K562 cells after treatment with indicated inhibitors for 72 h.

Flt3-negative cell line with Mk differentiation potential, K562, was used to evaluate the effects of gilteritinib. Flt3 inhibitors can be classified into type 1 and type 2. Type 1 inhibitors interact with the active conformation and are able to inhibit both Flt3-ITD and Flt3-TKD mutations, while type 2 inhibitors interact with the inactive conformation thus prevent activation, and do not inhibit Flt3-TKD mutations (17). As gilteritinib, a type 1 inhibitor, can inhibit the tyrosine kinase AXL, which is implicated in Flt3 inhibitor resistance (12, 18), type 2 inhibitor quizartinib, and AXL inhibitor dubermatinib were included in our screening (schematic diagram showing targets of drug action in **Figure 2A**). We initially evaluated the effects on cell proliferation, Mk differentiation, and cellular apoptosis in wild-type K562. Selected concentration was based on reported 50% inhibitory concentration (IC_50_). We found that PMA and dubermatinib markedly reduced cell proliferation while gilteritinib and quizartinib did not (**Figure 2B**). This finding is consistent with previous reports that PMA treatment leads to prolonged activation of the MAPK pathway, which is required for PMA-induced growth arrest and megakaryocytic differentiation (19–21). We further evaluated whether dubermatinib triggers differentiation or induces cellular apoptosis. Flow cytometric analysis of Annexin V showed that dubermatinib induced cellular apoptosis similar to PMA (**Figure 2C**). Dubermatinib also induced a modest shift in CD41a and CD61 fluorescent activity (**Figure 2D**). Interestingly, a megakaryocyte morphology was evident on cytospins from dubermatinib and PMA treated cultures, including increase in cell size and the presence of polylobate nuclei (**Figure 2E**).

### Effects of Flt3-ITD on Cellular Response to Inhibitors and Recovery of Gilteritinib Sensitivity

**Figure 3:**
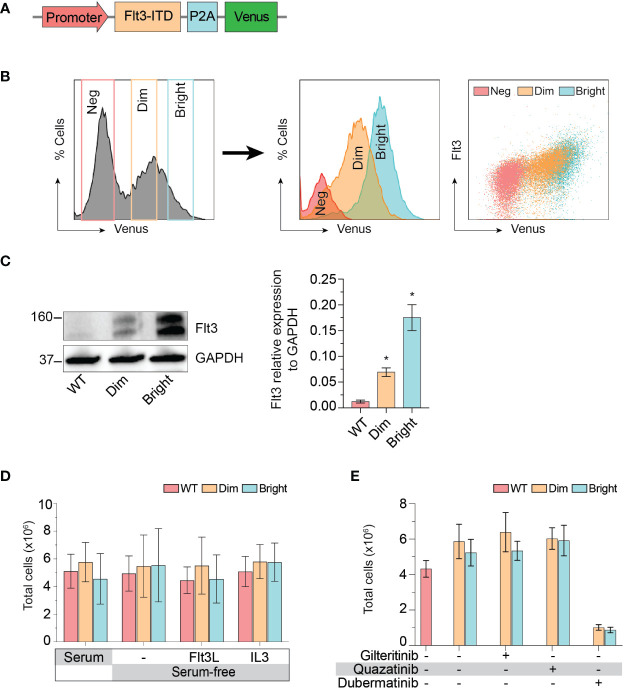
Effect of Flt3-ITD on K562 response to inhibitors. **(A)** Schematic diagram of Flt3-ITD overexpressing cassettes. **(B)** Flow cytometric histogram showing gating strategy used for fluorescent-activated cell sorting. Sorted cells were analyzed several weeks after expansion and showed stably expressed Flt3. Neg, negative. **(C)** Western blot showing total Flt 3 protein expression from wild type, dim, and bright K562. Representative pictures from three independent experiment are shown. Bar graph showing mean±SD of Flt3 protein expression relative to GAPDH. * indicates significance compared with wild type. **(D)** Cell proliferation profile of wild type, dim, and bright K562 cultured under indicating conditions for 72 h. Bar graph showing mean±SD from three independent experiments performing in parallel. Flt3L, Flt3 ligand; IL3, interleukin 3. **(E)** Cell proliferation profile of wild type, dim, and bright K562 treated with indicating 4 nM gilteritinib, 8 nM quizartinib, and 100 nM dubermatinib for 72 h. Bar graph showing mean ± SD from three independent experiments performing in parallel.

**Figure 4:**
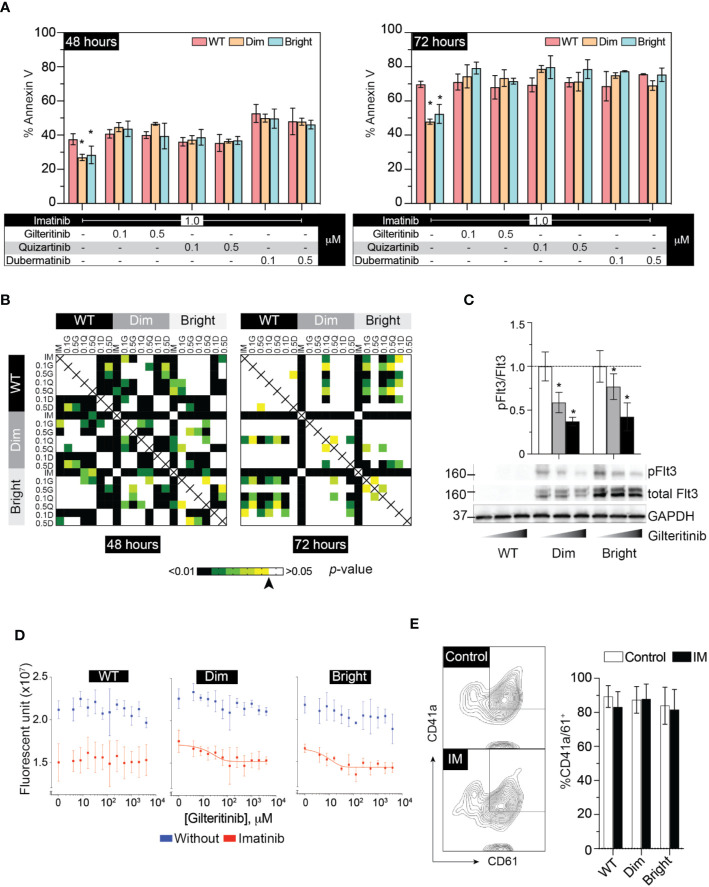
Flt3 mutant protect K562 from IM-induced apoptosis and IM recovers sensitivity to gilteritinib. **(A)** Flow cytometric analysis of Annexin V and 7AAD following 48–72 h treatment under the indicated condition. Results from three independent experiments are shown as mean ± SD. * indicates significance. **(B)** Heatmap showing *p*-value of two-way ANOVA followed by multiple comparison test from apoptosis assay by controlling the False Discovery Rate. Arrow indicates *p* value = 0.05. **(C)** Western blot analysis of pFlt3(y842), total Flt, and GAPDH following gilteritinib treatment. **(D)** Gilteritinib concentration-response curve showing proliferation of wild type, dim, and bright K562 after removing *Bcr/Abl1* translocation advantages. Cells were treated with 1 μM imatinib in a low-serum condition for 24 h. Viable cells were analyzed using Cell Titer Blue assay. Results are shown as mean ± SD from three independent experiments in duplicate. When possible, non-linear sigmoidal curve fit using GraphPad Prism are shown. **(E)** Flow cytometric analysis of CD41a/CD61 after treatment with imatinib. Results from three independent experiments are shown as mean ± SD. IM, imatinib.

The cellular mitogenic activity of Flt3-ITD is regulated by SOCS6 (suppressor of cytokine signaling 6) through direct binding to phosphorylated tyrosines 591 and 919 of Flt3. The presence of SOCS6 enhances ubiquitination of Flt3 and promotes degradation (22), while its absence promotes transformation of cells by Flt3-ITD *in vitro*. We therefore used Flt3-ITD with Y591F/Y919F double mutation to maximize its oncogenic effects. We tagged Flt3-ITD with Venus reporter using self-cleavage peptide P2A (**Figure 3A**) to generate stably expressed cell line. This approach allows the selection of differentially expressed, low and high Flt3-ITD population based on fluorescent activity. Stably transfected cells were enriched by fluorescent-activated cell sorting into Flt3-ITD^dim^ and Flt3-ITD^bright^ population (**Figure 3B**). Long-term culture of the sorted cells persistently demonstrated different levels of Flt3-ITD expression (**Figures 3B, C**). *In vitro* and murine studies demonstrated that constitutively active Flt3 mutants allow acute myeloid leukemia cells to grow in the absence of interleukin 3 (IL-3) (23–26). Thus, we included serum free-conditions supplemented with either Flt3 ligand or IL-3 to our assay. Cells were cultured in complete medium, serum-free condition, and serum-free condition supplemented with either Flt3L or IL3. However, we found that Flt3-ITD did not affect cell proliferation of K562 regardless of conditions (**Figure 3D**). Similarly, treatment with gilteritinib or quizartinib did not affect cell proliferation, whereas dubermatinib retained its antiproliferative effect (**Figure 3E**). Given that K562 was derived from chronic myeloid leukemia patient, the *Bcr/Abl1* translocation present in the genetic background potentially could alter the biology of Flt3 in our findings. Thus, we added the *Bcr/Abl1* inhibitor imatinib at 1 μM per previous reports from our group (27, 28) to disable its oncogene advantage. Overexpressed Flt3-ITD provided survival advantage to K562 by protecting it from imatinib-induced apoptosis, but not additional proliferative activity. Gilteritinib and quizartinib did not induce additional apoptosis at either 48 or 72 h (**Figure 4A**). Two-way ANOVA with multiple comparison *post hoc* test indicated that both Flt3-ITD^dim/bright^ K562 are susceptible to all inhibitors (compared with imatinib treatment, **Figure 4B**). Western blot analysis on phosphorylated tyrosine at 842 (pY842) confirmed kinase activity of overexpressed Flt3-ITD in the cells. Gilteritinib at 4 nM is sufficient to inhibit Flt3 phosphorylation (**Figure 4C**). Cell Titer Blue assay demonstrated that addition of imatinib recovered a sensitivity to gilteritinib in both Flt3-ITD^dim^ and Flt3-ITD^bright^ K562, but not in wild type K562 (**Figure 4D**).

### Megakaryocytic Differentiation

**Figure 5:**
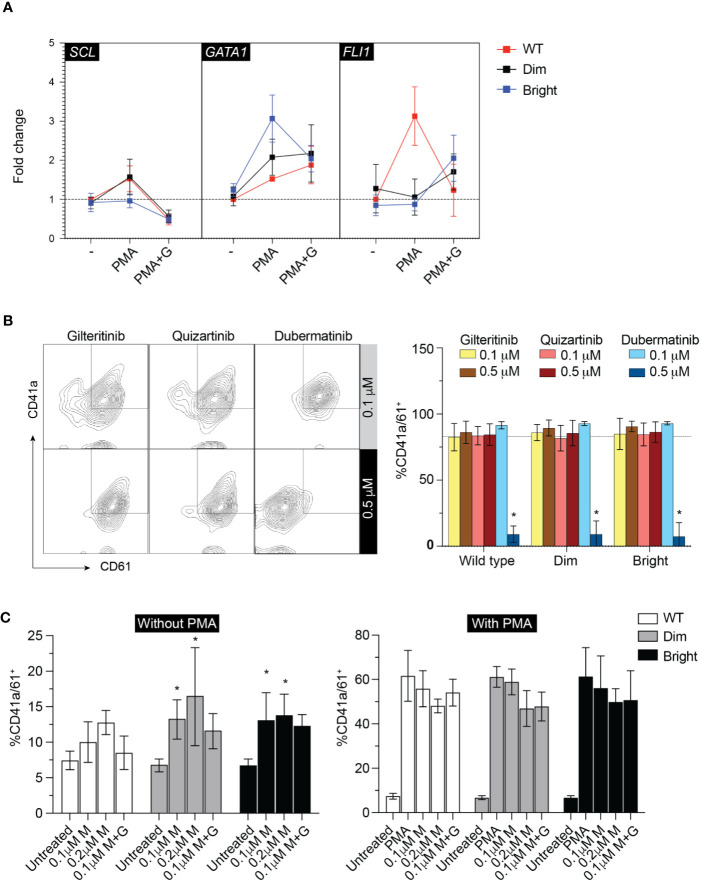
Megakaryocytic differentiation. **(A)** qPCR showing *GATA1*, *SCL*, *FLI1* after treatment with 0.1 µM gilteritinib for 24 h. G, gilteritinib. **(B)** Flow cytometric analysis of CD41a/CD61 after treatment with indicated inhibitor for 48 h. Results from three independent experiments are shown as mean ± SD. * indicates significance. - = imatinib treatment; WT, wild type. **(C)** Flow cytometric analysis of CD41a/CD61 after treatment with indicated inhibitor for 48 h. Results from three independent experiments are shown as mean ± SD. * indicates significance. M, midostaurin; G, gilteritinib.

To evaluate the effect of Flt3-ITD on Mk differentiation, wild-type, Flt3-ITD^dim^, and Flt3-ITD^bright^ K562 were treated with inhibitors for 48 h under low-serum concentration to maximize the effect of inhibitors while maintaining cell viability. Added imatinib did not affect Mk differentiation in all cell groups (**Figure 4E**). Quantitative PCR of megakaryocytic transcription factors including *GATA1*, *FLI1*, and *SCL* showed increased expression following PMA treatment and tendency to decrease with gilteritinib treatment (**Figure 5A**). Flow cytometric analysis of Mk differentiation using CD41a and CD61 showed gilteritinib and quizartinib did not affect percentage of CD41a^+^61^+^ in any groups, while dubermatinib at high doses decreased Mk (**Figure 5B**). We also found midostaurin can modestly induce Mk differentiation, though this effect diminished in the presence of PMA (**Figure 5C**).

## Discussion

The phase III ADMIRAL trial showed improved overall survival with the gilteritinib *vs* salvage chemotherapy in patients with Flt3-mutated acute myeloid leukemia (13), making gilteritinib a routine therapy for adult AML patients who have relapsed or refractory diseases with a Flt3 mutation. We followed six patients receiving gilteritinib and found marked expansion of megakaryocytes in a subset, which has not yet been reported. Flt3 expression is associated with Erythroid (E)-Mk development (29, 30). Lin^−^Sca1^+^kit^+^Flt3^+^ HSCs are highly proliferative and show lymphoid or myeloid differentiation potential, but lack E-Mk potential. Treatment with Flt3 inhibitor also promotes significantly increased platelet-like particle production from MK cell line (5). To address the association of Flt3 and Mk development, we reviewed bone marrow biopsy from patient with and without Flt3 mutation and found that patients with Flt3 mutants showed statistically higher numbers of megakaryocytes in post-therapy marrows. However, midostaurin was identified as a potential confounder as it was part of the treatment for Flt3-mutated AML in most patients and have been shown to induce Mk differentiation in human leukemia cells (16). Thus, to explore whether gilteritinib can directly promote Mk differentiation, we assessed it and other associated agents (quizartinib and dubermatinib) effects and on Mk production from wild-type K562 and K562 overexpressing double mutated Flt3-ITD^Y591F/Y919F^. As expected, neither of the small molecule inhibitors induced Mk differentiation in the wild-type Flt3-negative K562. Unexpectedly, AXL inhibitor dubermatinib induced apoptosis and Mk differentiation in K562. A marked reduction in CD41a^+^61^+^ (**Figure 5B**) at higher doses of dubermatinib was likely due to significant apoptosis (**Figure 2C**). We found overexpression of Flt3-ITD did not provide additional proliferative advantage to the full-blown blastic phase leukemia K562 line or additional Mk differentiation propensity. However, inclusion of imatinib in all the downstream assay to remove *Bcr/Abl1* oncogenic effects recovered gilteritinib susceptibility. Flt3-ITD protected Flt3-ITD^dim^ and Flt3-ITD^bright^ K562 from imatinib-induce apoptosis while addition of gilteritinib and quizartinib removed its antiapoptotic activity. Since Flt3-ITD activates multiple downstream targets involved in the PI3K/AKT, RAS/mitogen-activated protein kinase (MAPK) pathways, and activator of transcription 5 (STAT5), this finding of resistance to apoptosis is consistent with previous report (31). Since almost all Flt3-mutated AML patients previously received midostaurin, we included midostaurin into our assays. We found that midostaurin significantly induced Mk differentiation in Flt3-ITD^dim^ and Flt3-ITD^bright^ K562. Wild-type K562 showed a tendency to differentiate into Mk but did not achieve statistical significance, indicating that midostaurin acted in concert with Flt3 mutants to promote Mk differentiation. This finding supported our observation of increased Mk in the Flt3-mutated AML patients. It also implied that interactions between chaperone proteins, which are apparently missing in our gilteritinib assays, and Flt3 are required to observe such effects. In contrast, dubermatinib strongly inhibited cell proliferation and induced apoptosis regardless of Flt3 status.

**Figure 6:**
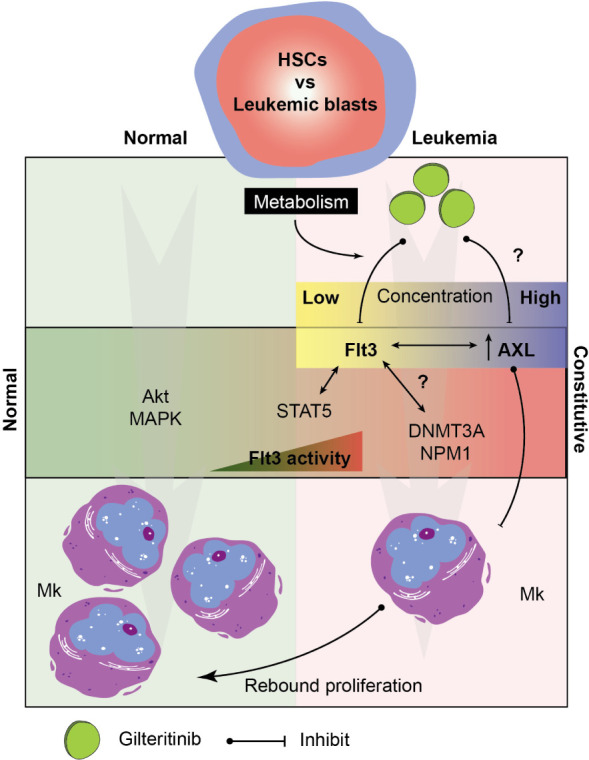
Hypothetical model of the signaling pathway involving in megakaryocytic differentiation in normal and leukemic marrow. Multifactorial model showing key players in the Mk development under normal and leukemic hematopoiesis. Drug metabolism determines tissue level of gilteritinib in each patient. Due to an extremely high variability of metabolic enzymes, genetic polymorphisms, drug interactions, and diets, a wide range of gilteritinib tissue concentration is expected. Gilteritinib at low concentration selectively inhibit constitutive Flt3 activity, but not AXL. At high concentration, gilteritinib inhibits increased AXL in leukemia patients disinhibiting its Mk inhibitory activity. Interactions between constitutive Flt3 activity, STAT5 activation, transcription factor (NMP1), and epigenetic modifications (DNMT3A) could impact Mk differentiation through Flt3/AXL crosstalk. HSCs, hematopoietic stem cells; Mk, megakaryocyte.

Recent clinical observation has described two distinct marrow responses, i.e. with and without differentiation, following gilteritinib treatment (14). Both patterns can show relatively stable total marrow cellularity to hypocellular marrow, but numbers of E and Mk were unaffected or showed relative hypoplasia. In contrary, rebound erythroid differentiation was also reported in some cases (32). While the mechanistic underpinnings of such diversified reports remain unclear and is likely multifactorial, it is worth noting that the pharmacokinetic profile of gilteritinib is notable for its large volume of distribution (Vd) with the population estimated central and peripheral Vd of 1,092 and 1,100 L, respectively. This profile indicates that gilteritinib distributes and accumulates within the tissue including bone marrow to a much higher level compared to plasma. In addition, gilteritinib is primarily metabolized by CYP3A4, which is notable for its extremely high variability (>100-fold) in the population due to polymorphisms, drug interactions, and diets (reviewed in Klein et al. (33)). Variables in tissue concentration of gilteritinib potentially plays an important role in the biphasic responses through its impactive AXL off-target inhibition. AXL, a member of phosphatidylserine-sensing receptor tyrosine kinases, the TAM family (Tyro3, AXL, and Mer), is significantly up-regulated in AML patient samples (34). Mice lacking all three receptors had impaired hemostasis and thrombocytopenia potentially due to platelet dysfunction and defective megakaryopoiesis (35). In fact, no prior studies demonstrated that pharmacological blockade of AXL signaling induced Mk differentiation. To the best of our knowledge, our results showing for the first time that AXL inhibitor is able to induce Mk differentiation suggesting the rebound proliferation when gilteritinib exhibits AXL inhibition disinhibiting high AXL levels in the AML patients (**Figure 6**). AXL transforming properties have been identified as a potential therapeutic target for AML (12, 36) and have been implicated in the resistance to quizartinib seen in Flt3-mutated AML *via* the STAT5 pathway. Since red blood cells and megakaryocytes arise from the megakaryocyte-erythroid progenitor and share many regulators including the transcription factors during hematopoiesis (37), our findings are likely similar to a report by Yun et al. (32).

Hyperactive Flt3-ITD activates downstream STAT5A/B transcription factors, which regulate gene expression in conjunction with other transcriptional regulators and epigenetic modifiers such as EZH2, TET1/2, and DNMT3A (reviewed in Wingelhofer et al. (38)). In our study, all patients with increased Mk shared *NPM1* and *DMMT3A* mutations, and might be, in part, associated with the expansion of Mk. Noticeably, residual disease persisted in all patients in this group regardless of transplantation. It is plausible that expanded Mk are derived from the leukemic blasts, therefore, the treatment-induced differentiation may complicate the definition of complete remission and its clinical significance.

Overall, our study showed that gilteritinib and the levels of Flt3-ITD do not directly affect Mk differentiation *in vitro*; however, their potential impact cannot be entirely excluded. The interaction between Flt3, AXL, and DNMT3A was highlighted as a plausible crosstalk mechanism that indirectly dictates Mk differentiation. Taken together, we propose a model showing possible signaling targets involving in the process (**Figure 6**). Further investigations in a larger population or animal models focusing on the role of AXL in the pathophysiologic process of Flt3-mutated leukemia is warranted its significance on clinical outcomes.

## Data Availability Statement

The original contributions presented in the study are included in the article/**Supplementary Materials**. Further inquiries can be directed to the corresponding author.

## Ethics Statement

The studies involving human participants were reviewed and approved by Institutional Review Board of the University of Wisconsin Madison. Written informed consent for participation was not required for this study in accordance with the national legislation and the institutional requirements.

## Author Contributions

KS designed, conducted the experiments, and wrote the manuscript. HJ conducted the Western blot analysis. YC conducted the cell proliferation assay and flow cytometry. AM conducted the quantitative PCR. SS evaluated the marrow biopsy. ER and IS conceptualized and supervised all aspects of the studies. All authors contributed to the article and approved the submitted version.

## Conflict of Interest

The authors declare that the research was conducted in the absence of any commercial or financial relationships that could be construed as a potential conflict of interest.
