# Supplementary material for: Megakaryocytic Expansion in Gilteritinib-Treated Acute Myeloid Leukemia Patients Is Associated With AXL Inhibition
Source: Front Oncol. 2020 Dec 9;10:585151. doi: 10.3389/fonc.2020.585151 (PMC7756118; doi:10.3389/fonc.2020.585151)
Supplement: Supplementary file 1 [file Table_1.docx]

**Supplemental figure 1**


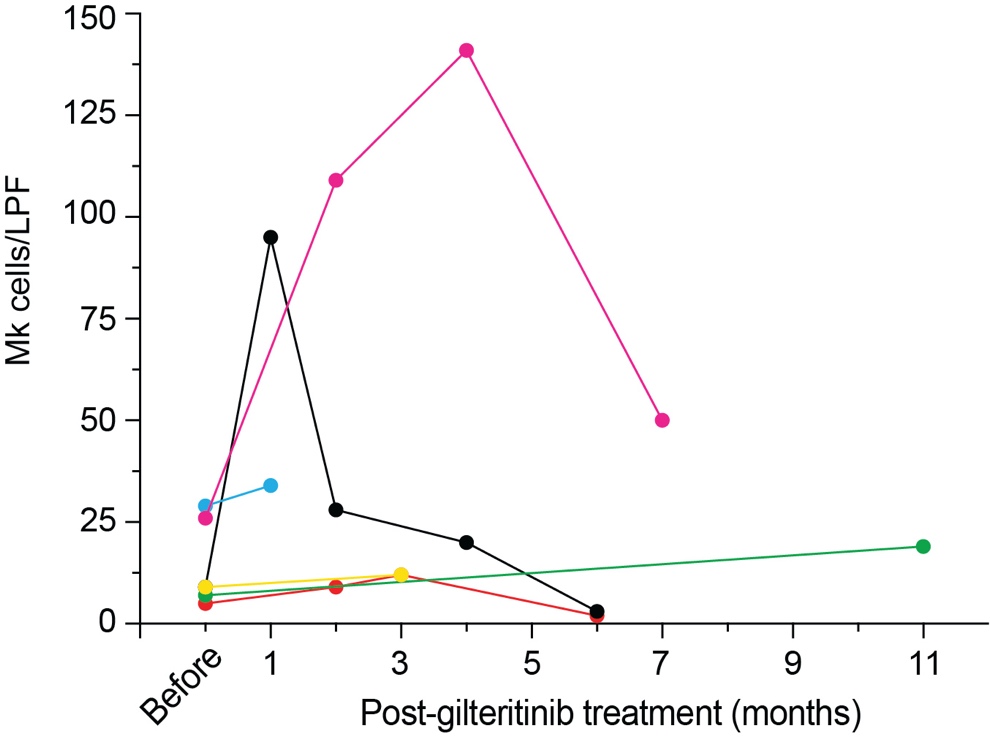


Timecourse of megakaryocytes following gilteritinib treatment. Each line graph represents individual patient corresponding to Figure 1E.
